# Sarcopenia in patients with hip fracture: A multicenter cross-sectional study

**DOI:** 10.1371/journal.pone.0184780

**Published:** 2017-09-13

**Authors:** Ole Martin Steihaug, Clara Gram Gjesdal, Bård Bogen, Målfrid Holen Kristoffersen, Gunhild Lien, Anette Hylen Ranhoff

**Affiliations:** 1 Kavli Research Centre for Geriatrics and Dementia, Haraldsplass Deaconess Hospital, Bergen, Norway; 2 Department of Clinical Science, University of Bergen, Bergen, Norway; 3 Department of Rheumatology, Haukeland University Hospital, Bergen, Norway; 4 Bergen University College, Bergen, Norway; 5 Department of Orthopedics, Haukeland University Hospital, Bergen, Norway; 6 Department of Rheumatology, Diakonhjemmet Hospital, Oslo, Norway; Garvan Institute of Medical Research, AUSTRALIA

## Abstract

**Background:**

Sarcopenia is prevalent in older persons and is a risk factor for falls, fractures, and mortality. The aim of this study was to determine a) the feasibility of determining sarcopenia in patients with acute hip fracture, b) the prevalence of sarcopenia and c) associations of sarcopenia with nutritional status and comorbidities.

**Methods:**

A multicenter cross-sectional study on sarcopenia in male and female patients with acute hip fracture. Participants were previously ambulatory and living in the community. Sarcopenia was assessed postoperatively with muscle mass estimated by anthropometry using triceps skinfold, arm circumference, height, weight and sex. Grip strength was measured by Jamar dynamometer and pre-fracture mobility was by self-report using the New Mobility Score.

**Results:**

Out of 282 patients, 202 were assessed for sarcopenia of whom 74 (37%) were diagnosed as sarcopenic. Sarcopenia was associated with age, odds ratio (OR) 1.4 per 5 years, 95% confidence interval (CI) [1.1, 1.8], ASA Physical Status Classification System score, OR 2.3 per point, 95% CI [1.3, 4.3] and number of medications at discharge, OR 1.2 per medication, 95% CI [1.0, 1.3] and inversely associated with BMI, OR 0.8, 95% CI [0.7, 0.9] and serum albumin, OR 0.9, 95% CI [0.8,1.0].

**Conclusions:**

Thirty-seven percent of assessed subjects were diagnosed with sarcopenia. Our data demonstrates that the prevalence of sarcopenia is associated with older age, malnutrition and comorbidities. Determining sarcopenia at the bedside was feasible in postoperative hip fracture patients by using grip strength, estimation of muscle mass by anthropometry and self-reported mobility.

## Introduction

Sarcopenia is a syndrome characterized by reduced muscle mass and reduced muscle function and an increased risk of disability and death [1]. Sarcopenia has recently been recognized as an independent condition with an International Classification of Disease Code [2]. Sarcopenia is a well-known risk factor for both falls and fractures: Reduced muscle strength makes it more difficult to regain lost balance and decreases the mechanical loading of the skeleton leading to reduced adaptive bone remodeling [3, 4]. Half of all hip fracture survivors will develop permanent impairments in mobility and 10–20% will become institutionalized [5]. The prevalence of sarcopenia in hip fracture patients is 17–74%, depending on population and definition of sarcopenia [6–8]. Norway has one of the highest rates of hip fracture in the world [9] and it is estimated that 4–5% of all deaths in the Norwegian population aged 50+ are attributable to hip fractures [10]. The reasons for this are unknown and sarcopenia is a candidate for explaining some of this excess risk. There has been only moderate improvement in the outcomes of patients with hip fracture since the 1960s [11]. Studies indicate that better outcomes are possible by involving geriatricians and increasing the intensity of rehabilitation efforts [12, 13]. Exercise and nutritional interventions are important interventions for sarcopenia [14] and in rehabilitation after hip fracture [15]. Smoking cessation and reduction of harmful alcohol intake can possibly reduce sarcopenia [16–18] and the risk of hip fracture [19–22]. There is no consensus on how sarcopenia should be operationalized. The three main methods are: low muscle mass as recommended by Janssen et al [23], low muscle mass with one of reduced physical performance or muscle strength, as recommended by the European Working Group on Sarcopenia in Older People (EWGSOP) [1] and low muscle mass and low grip strength, as recommended by the Foundation for the National Institutes of Health Biomarkers Consortium Sarcopenia Project [24]. These recommendations are based on studies on older people living in the community. Investigating the feasibility of determining sarcopenia in acute hip fracture patients is necessary before assessment of sarcopenia can be introduced in clinical practice. The aims of this study are to:

1. assess the feasibility of determining sarcopenia in acute hip fracture patients.
2. determine the prevalence of sarcopenia and investigate how sarcopenia is associated with risk factors for adverse clinical outcomes: older age and male sex [10], nutritional risk and low albumin, low vitamin D, low body mass index (BMI) [25], comorbidities and polypharmacy, and impairments in activities of daily living [26].
3. Investigate the separate associations of muscle mass, grip strength and mobility with the same risk factors.

## Materials and methods

This is a cross-sectional study on sarcopenia in patients with hip fracture. Patients were included in the immediate postoperative period at three hospitals in Norway, 2011–2013. Patients eligible for participation were 65 years or older, ambulatory before the hip fracture and willing to provide written informed consent. Permanent residents of nursing homes, patients who were medically unstable or had a life expectancy of less than 3 months, were excluded. Collection of data was by the authors or research personnel, with different teams at the different hospitals. All personnel received training and guidance from the first author (OMS). Research staff were not present on the wards at all times, such as during weekends or holidays. Pre-fracture independence in activities of daily living (meals, bathing, grooming, dressing, continence, toileting, transferring and ambulation) was determined by the modified Barthel index (B-ADL), a summary score with a range 0–20 [27]. Nutritional risk was assessed using the Nutritional Risk Screening Score 2002 (NRS 2002) [28]. The NRS 2002 is a screening tool for identifying hospitalized patients likely to benefit from nutritional interventions. It is scored 0–3 points for nutritional state, 0–3 points for illness severity and an additional point if aged >70 years, for a total of score 0–7. Patients with hip fracture will typically be given one point for illness severity. Serum albumin and 25-OH vitamin D was measured in the fasting state, preoperatively at one hospital and postoperatively at two hospitals. Participants use of supplemental vitamin D (as tablets or cod liver oil) was determined by food frequency questionnaire and chart review. Comorbidities were assessed by chart review for determining the Charlson index [29]. The Charlson index is a list of chronic diseases, weighted by severity, that has been found to predict mortality. The American Society of Anesthesiologists Physical Status Classification System (ASA) score is a grading system of the preoperative health of surgical patients, range 1–5 [30]. The number of medications used regularly and as needed was assessed at discharge.

### Determining sarcopenia

Participants were identified as being “not sarcopenic” or “sarcopenic” using the criteria recommended by the EWGSOP [1]. To be categorized as sarcopenic participants had to have low muscle mass and one of either low grip strength or low mobility. Total body muscle mass was estimated by anthropometry by the method of Heymsfield et al. [31] using gender, height, arm circumference and triceps skinfold. Arm circumference was measured on the right arm at the mid-point between the acromion and olecranon process with the arm hanging down. Triceps skinfold was measured on the posterior aspect of the same arm at the same level using a skinfold caliper (Harpenden, Baty International, Great Britain). Measurements were repeated until two readings were within 1mm. The values for total body muscle mass were converted to appendicular lean mass (ALM) using model 1 of Kim et al [32]. It has previously been reported, using data from this study, that anthropometry by the Heymsfield method was able to identify patients with low muscle mass, compared to Dual Energy X-ray absorptiometry (DXA) [33]. Patients were weighed in light clothing using the scales available on the hospital wards. Height was measured by wall-mounted stadiometer, except for a few cases where the patient was unable to stand. In those cases, height measured at other time-points, self-reported height or the distance from heel to crown while lying in bed was used. Cut-points for low muscle mass were chosen based on the recommendations of the EWGSOP [1], ALM divided by height squared, ≤7.25 kg^.^m^-2^ for men and ≤5.67 kg^.^m^-2^ for women.

Grip strength was measured three consecutive times in one hand and immediately afterward on the other hand with a Jamar Hydraulic Dynamometer (Sammons Preston, USA) while the patient was sitting in bed or on a chair, with the elbow flexed, the wrist in the neutral position, and with verbal encouragement. There was a brief interval between attempts while the dynamometer was repositioned. The single best value of all six measurements was used. Low grip strength was defined as ≤ 30kg for men and ≤ 20kg for women, as recommended by the EWGSOP[1]. Grip strength and muscle mass was determined at daytime in the mainly bed-bound participants without consideration of meals, recent physical activity, bladder voiding or hydration.

Mobility was determined by the New Mobility Score (NMS). The NMS assesses mobility in the two weeks prior to the fracture by interview. It ranges 0–9; a score of zero indicates that the person is not ambulatory and nine indicates an ability to walk without assistance while shopping. The NMS predicts physical performance and mortality after hip fracture [34–36]. Low mobility was defined as NMS <5, based on the cut-point recommended for predicting mortality after hip fracture [34]. Anthropometry for determining muscle mass was chosen because it is an established technique for determining body composition [37], is in common use [38], inexpensive and more easily performed on patients with pain on mobilization compared to DXA. Estimating muscle mass by anthropometry in patients with acute hip fracture requires some effort, mainly in determining height and weight. Grip strength is quickly measured, but requires an alert patient able to take instruction and with reasonable hand function. The NMS was chosen because it was assumed that some participants would be unable to perform tests of physical performance and because self-reported mobility has been found to have similar psychometric and predictive properties to objective tests [39]. Mobility by the NMS can be determined in a minute in a bed-bound patient, as long as the patient or proxy is able to answer questions about pre-fracture mobility.

### Ethics

All participation was by written, informed consent. Patients were included in the postoperative phase as pain and anxiety is less after surgery. Participating hospitals, Haraldsplass Deaconess Hospital, Haukeland University Hospital and Diakonhjemmet Hospital, and the Regional Committee on Medical and Health Research Ethics approved the study (2011/1322/REK sør-øst B). The study was conducted according to the principles of the Declaration of Helsinki [40].

### Statistical analysis

Participants were described according to sarcopenia status with median and interquartile range (IQR) or mean and standard deviation (SD) and the differences between groups were analyzed with the Mann–Whitney–Wilcoxon test. The association between sarcopenia as dependent variable and the different risk factors as independent variables was analyzed in separate logistic regression analyses with odds ratios (OR) and 95% confidence intervals (95% CI). Separate analysis was also performed with low muscle mass, low grip strength and low mobility as dependent variables. Regression analyses were adjusted for age, sex and BMI and analysis of vitamin D was additionally adjusted for using supplemental vitamin D. Age, sex and BMI were included in the models because they are established associations of grip strength and muscle mass [41]. P≤0.05 was considered significant. Analysis was by Stata 14.0 (Stata Corp., USA)

## Results

**Fig 1:**
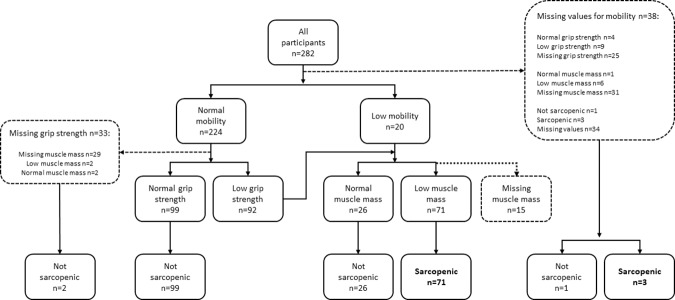
Participants assessed for mobility, grip strength and muscle mass.

Of the included 282 patients with acute hip fracture, low muscle mass was found in 61% (118/194), low grip strength in 52% (116/222), and low mobility in 8% (20/244). Sarcopenia prevalence was 37% (74/202). Fig 1 illustrates which participants were assessed for mobility, grip strength and muscle mass. Participants with sarcopenia were older, had evidence of nutritional risk as indicated by lower BMI, lower albumin and higher scores on the NRS 2002. Participants with sarcopenia were also characterized by longer hospital stay, higher ASA score at operation, used a greater number of medications at discharge and had more impairments in activities of daily living before the hip fracture (Table 1). Participants with missing values for sarcopenia status had lower B-ADL scores compared to those who did not have missing values, and women with missing values had lower grip strength and mobility. Reasons for missing values included patients being too ill for or refusing specific examinations, or that they were discharged before the data collection was completed. Intracapsular fractures constituted 59% of the fractures. For 8% of patients this was their second hip fracture. Grip strength and ALM were determined at a median of 4 days after surgery, the interquartile range was 3 to 6 days, and the total range was from the day before surgery to 24 and 34 days after surgery, respectively. Supplemental vitamin D was used by 50% and was associated with a significantly higher serum vitamin D, 63 (SD 26) versus 47 (SD 23) 10^−6^ mol^.^m^-3^ (nmol/L). During the period of inclusion, 1592 patients were admitted for hip fracture surgery at the three hospitals.

**Table 1:** Characteristics of participants by sarcopenia status.

	Not sarcopenic, n = 128			Sarcopenic, n = 74		
Age, years [IQR]	77.5	[70,5–85]	n = 128	82	[76–86]	n = 74**
Female, N (%)	99	(77)	n = 128	53	(72)	n = 74
Length of hospital stay, days [IQR]	6	[5–8]	n = 128	7.5	[6–11]	n = 74**
BMI, kg^.^m^-2^ [IQR]	25.3	[22.7–28.0]	n = 110	22.0	[19.5–24.2]	n = 67**
NRS 2002 [IQR]	2	[2–2]	n = 117	2	[2–3]	n = 70**
Albumin, kg^.^m^3^ (SD)	35.9	(5.2)	n = 113	32.6	(6.3)	n = 59**
User of vitamin D supplement, N (%)	60	(50)	n = 119	31	(46)	n = 56
Vitamin D, 10^−6^ mol^.^m^-3^ [IQR]	53	[34–75]	n = 111	48	[31–66]	n = 64
Charlson index [IQR]	0.5	[0–1]	n = 128	1	[0–2]	n = 74
ASA score [IQR]	2	[2–3]	n = 128	3	[2–3]	n = 74**
Medications, number [IQR]	7	[5–10]	n = 128	8	[7–11]	n = 73**
B-ADL [IQR]	20	[19–20]	n = 88	20	[18–20]	n = 57*
ALM^.^height^2^, kg-^.^m^-2^ [IQR]	6.4	[5.6–7.4]	n = 115	4.7	[4.0–5.2]	n = 74**
ALM^.^height^2^ ─ Women, kg-^.^m^-2^ [IQR]	6.3	[5.6–7.1]	n = 90	4.4	[4.0–5.1]	n = 53**
ALM^.^height^2^ ─ Men, kg-^.^m^-2^ [IQR]	7.1	[6.1–7.8]	n = 25	5.1	[4.5–5.8]	n = 21**
Grip strength, kg [IQR]	25	[21–32]	n = 126	17	[12–20]	n = 74**
Grip strength ─ Women, kg [IQR]	22	[20–26]	n = 96	14	[12–18]	n = 53**
Grip strength ─ Men, kg [IQR]	40	[34–43]	n = 29	24	[20–27]	n = 22**
New Mobility Score [IQR]	9	[7–9]	n = 127	7	[5–9]	n = 71**
New mobility score–Women [IQR]	9	[7–9]	n = 98	7.5	[5–9]	n = 52**
New mobility score–Men [IQR]	9	[7–9]	n = 29	7	[5–9]	n = 19*

IQR: Values are medians and interquartile range. SD: Values are means and standard deviation.

* P≤0.05 or

**P ≤0.01 is the probability for difference by Mann–Whitney–Wilcoxon test.

BMI: Body mass index. ASA score: The ASA Physical Status Classification System. Barthel ADL: Barthel activities of daily living. ALM: Appendicular lean mass.

**Fig 2:**
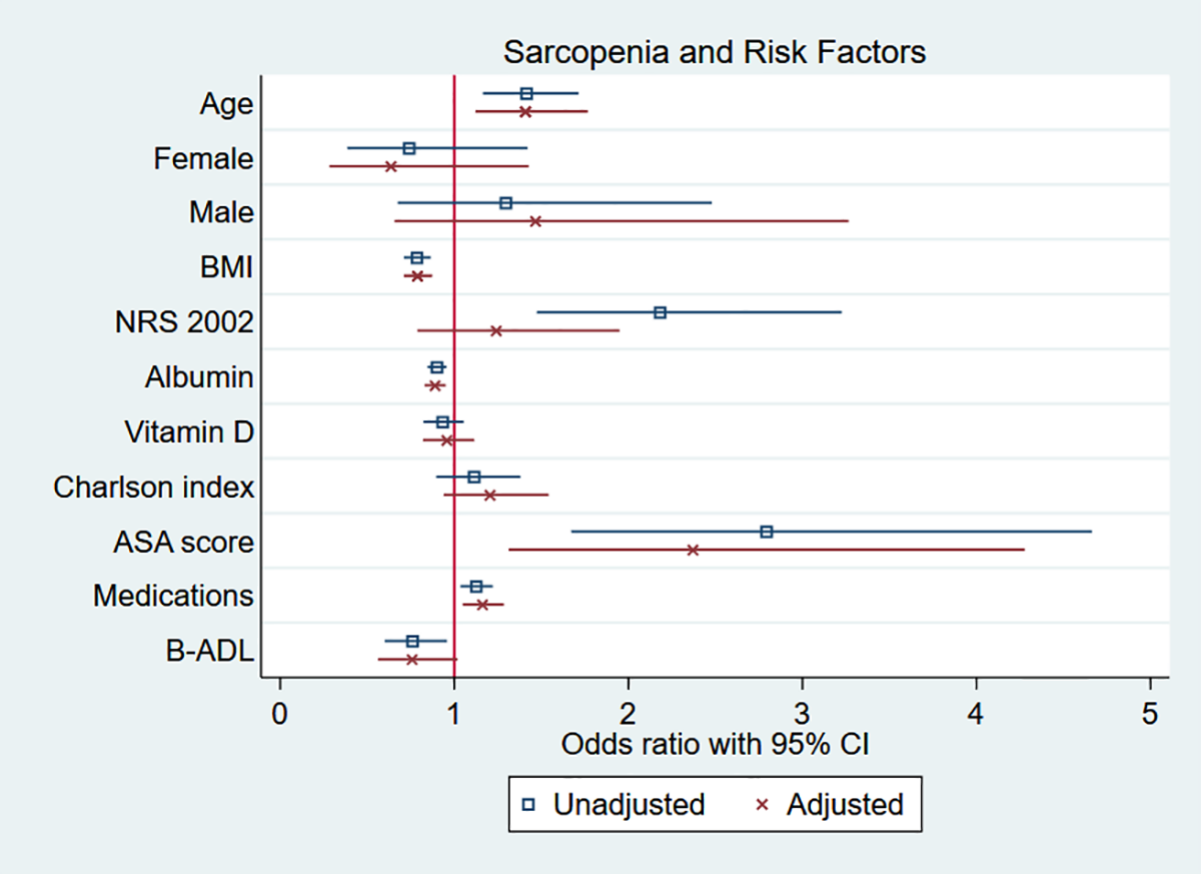
Sarcopenia and risk factors. Adjusted regression analysis using age, sex and BMI as covariates and the analysis of vitamin D additionally adjusted for being a user of supplemental vitamin D. Estimate for age is for 5-year increase and estimate for vitamin D is for increase of 10^−5^ mol^.^m^-3^ (10 nanomol/L).

The results of regression analysis for sarcopenia are presented in Fig 2 and Table 2. In adjusted analysis, sarcopenia was positively associated with age, OR 1.4 for each 5-year increase, 95% CI [1.1, 1.8], ASA score, OR 2.4, 95% CI [1.3, 4.3], and number of medications at discharge, OR 1.2, 95% CI [1.0, 1.3]. BMI, OR 0.8, 95% CI [0.7, 0.9] and serum albumin, OR 0.9 95% CI [0.8, 1.0], were associated with not having sarcopenia.

**Table 2:** Predictors of sarcopenia.

	Univariable analysis					Adjusted analysis				
	OR	[95% CI]	P-value	R^2^	n	OR	[95% CI]	P-value	R^2^	n
**Age, 5 years**	1.4	[1.2,1.7]	<0.001	0.05	202	1.4	[1.1, 1.8]	0.003	0.18	177
**Female**	0.7	[0.4, 1.4]	0.4	<0.01	202	0.6	[0.3, 1.4]	0.3	0.18	177
**Male**	1.4	[0.7, 2.6]	0.3	<0.01	202	1.6	[0.7, 3.5]	0.3	0.18	177
**BMI, kg**^.^**m**^**-2**^	0.8	[0.7, 0.9]	<0.001	0.13	177	0.8	[0.7, 0.9]	<0.001	0.18	177
**NRS 2002**	2.2	[1.5, 3.2]	<0.001	0.08	187	1.2	[0.8, 1.9]	0.4	0.19	168
**Albumin, kg**^.^**m**^**-3**^	0.9	[0.8, 1.0]	<0.001	0.06	172	0.9	[0.8, 1.0]	0.001	0.19	147
**Vitamin D, 10**^**−5**^ **mol**^.^**m**^**-3**^	0.9	[0.8, 1.1]	0.3	<0.01	175	1.0	[0.8, 1.1]	0.6	0.17	141
**Charlson index**	1.1	[0.9, 1.4]	0.3	<0.01	202	1.2	[0.9, 1.5]	0.1	0.18	177
**ASA score**	2.8	[1.7, 4.7]	<0.001	0.06	202	2.4	[1.3, 4.3]	0.004	0.21	177
**Medications**	1.1	[1.0, 1.2]	0.005	0.03	201	1.2	[1.0, 1.3]	0.004	0.22	176
**B-ADL**	0.8	[0.6, 1.0]	0.020	0.03	145	0.8	[0.6, 1.0]	0.066	0.25	139

Sarcopenia as dependent variable in separate logistic regression analyses. Adjusted analyses are with age, gender and BMI as covariates and for vitamin D as a predictor is additionally adjusted for being a user of supplemental vitamin D. OR: Odds ratio. CI: Confidence interval. R^2^: Adjusted R^2^. BMI: Body mass index. ASA score: the ASA Physical Status Classification System, points. B-ADL: Barthel activities of daily living. Vitamin D 10^−5^ mol^.^m^-3^ (10 nmol/L).

Results for the adjusted regression analyses for low muscle mass, low grip strength and low mobility are presented in Table 3. Low muscle mass, using sex-specific cut-points, was associated with male gender, OR 5.4, 95% CI [1.8, 15.8]. BMI and albumin were negatively associated with low muscle mass. Low grip strength was associated with increasing age and ASA score and associated with lower BMI and albumin. Low mobility was associated with age and number of medications at discharge and negatively associated with impairments in activities of daily living.

**Table 3:** Muscle mass, grip strength, mobility and risk factors.

	Low muscle mass					Low grip strength					Low mobility				
	OR	[95% CI]	n	P	R^2^	OR	[95% CI]	n	P	R^2^	OR	[95% CI]	n	P	R^2^
**Age, 5 years**	1.0	[0.8, 1.3]	170	0.97	0.27	1.6	[1.3, 2.0]	186	<0.001	0.15	1.5	[1.0, 2.2]	194	0.038	0.07
**Female**	0.2	[0.1, 0.6]	170	0.002	0.27	1.2	[0.6, 2.6]	186	0.6	0.15	0.6	[0.2, 2.2]	194	0.5	0.07
**Male**	5.4	[1.8, 15.8]	170	0.002	0.27	0.8	[0.4,1.7]	186	0.6	0.15	1.6	[0.4, 5.5]	194	0.5	0.07
**BMI, kg**^.^**m**^**-2**^	0.7	[[0.6, 0.8]	170	<0.001	0.27	0.9	[0.8, 1.0]	186	0.003	0.15	0.9	[0.8, 1.0]	194	0.2	0.07
**NRS 2002**	1.6	[0.8, 3.3]	163	0.2	0.27	1.1	[0.8, 1.7]	175	0.6	0.17	1.5	[0.9, 2.6]	182	0.1	0.10
**Albumin, kg**^.^**m**^**-3**^	0.9	[0.8, 0.9]	141	0.001	0.31	0.9	[0.9, 1.0]	154	0.006	0.17	1.0	[0.9, 1.1]	163	0.9	0.03
**Vitamin D, 10**^**−5**^ **mol**^.^**m**^**-3**^	1.0	[0.8, 1.1]	138	0.6	0.31	0.9	[0.7, 1.0]	147	0.059	0.17	0.9	[0.6, 1.2]	154	0.4	0.10
**Charlson index**	1.1	[0.8, 1.4]	170	0.7	0.27	1.1	[0.9, 1.4]	186	0.4	0.15	1.3	[0.9, 1.8]	194	0.2	0.09
**ASA score**	1.9	[1.0, 3.6]	170	0.052	0.29	1.8	[1.0, 3.0]	186	0.038	0.16	2.3	[0.9, 6.2]	194	0.09	0.10
**Medications**	1.0	[0.9, 1.2]	169	0.5	0.28	1.1	[1.0, 1.2]	185	0.08	0.16	1.2	[1.0, 1.5]	193	0.015	0.13
**B-ADL**	0.9	[0.6, 1.3]	131	0.5	0.31	0.8	[0.6,1.0]	148	0.052	0.19	0.5	[0.3, 0.7]	149	<0.001	0.34

Low muscle mass, low grip strength and low mobility as dependent variables in separate logistic regression analyses. All analyses are adjusted for age, gender and BMI and analyses for serum vitamin D are additionally adjusted for use of supplemental vitamin D. OR: Odds ratio. CI: Confidence interval. n: number of participants without missing values and available for analysis. P: Probability of association being random. R^2^: Adjusted R^2^. BMI: Body mass index. ASA score: the ASA Physical Status Classification System, points. B-ADL: Barthel activities of daily living. ALM: Appendicular lean mass. Low muscle mass ≤7.25 kg^.^m^-2^for men and ≤5.67 kg^.^m^-2^ for women, defined as appendicular lean mass divided by height squared. Low grip strength is ≤30kg for men and ≤20kg for women. Low mobility is NMS <5. Vitamin D 10^−5^ mol^.^m^-3^ (10 nmol/L).

## Discussion

The aims of this study were to determine the feasibility of identifying sarcopenia in acute hip fracture patients, estimate the prevalence of sarcopenia and the associations between sarcopenia and risk factors for adverse clinical outcomes after hip fracture. Two hundred and eighty-two participants were included and sarcopenia status was determined in 202. Determining sarcopenia status using the bedside methods of anthropometry, grip strength and the NMS was feasible, is relevant to clinicians as a bedside tool, and is possible to implement on busy hospital wards. The rate of 28% of unsuccessful assessments for sarcopenia indicates that further improvement is warranted. Feasibility would be improved if muscle mass could be estimated without determining height or weight. Malmstrom and Morley have recommended the simple screening tool SARC-F as method to diagnose sarcopenia [42]. The SARC-F is a questionnaire containing 4 questions about physical performance and one question about falls in the last year. Future research should investigate the role of the SARC-F in patients with hip fracture. In the present study, sarcopenia was determined by anthropometry, which is considered a less precise method compared to DXA or bioelectrical impedance analysis. Anthropometry is adequate at identifying low muscle mass [43] and is able to identify increased mortality risk in males [44, 45]. Because of pain and severe mobility impairment, it is difficult to measure muscle mass by DXA in patients with hip fracture. Compared to anthropometry, DXA is more expensive, requires bulky equipment, takes longer time to do, and requires more trained personnel. DXA estimates of muscle mass are sensitive to acute changes in muscle water content, such as seen with changes in muscle glycogen or creatinine due to feeding or dehydration [46].

A strength of the present study is that participants were recruited at three separate hospitals. This reduced the influence of investigator specific factors such as confirmation bias and reduced the influence of hospital related factors such as differences in quality of care, differences in catchment populations, or selective recruitment processes. A multi-center study is leads to greater generalizability of results. A weakness of the multi-center design was less control of the conduct of the study, leading to loss of information on participants who were screened but not included and missing values.

Three recent cross-sectional studies have investigated sarcopenia in acute hip fracture patients using the EWGSOP framework. There was great variation in the prevalence of sarcopenia, 12–74% in men and 18–68% in women [6–8]. This variation in the prevalence of sarcopenia is likely due to differences in patient groups, measurement techniques and the use of different cut-points. The studies were from Hong Kong, Spain and Italy. Ho et al studied Chinese patients with hip fracture and used lower cut-points for grip strength at <26kg for men and <18kg for women. Muscle mass was determined by DXA a mean 14 days after the fracture. In the study by Gonzalez-Montalvo et al sarcopenia was determined before surgery. Muscle mass was determined by bioelectrical impedance and the cut-point for low muscle mass was higher than in the present study, at <6.68 kg∙m^-2^ in women and <8.31 kg∙m^-2^ in men. The study by Di Monaco et al was on Italian women selected to undergo intensive rehabilitation after hip fracture. Di Monaco et al found that the Barthel index at the start of rehabilitation was lower in the group with sarcopenia, while both the study by Gonzalez-Montalvo et al. and Di Monaco et al. reported an association between low BMI and sarcopenia [6, 8].

Compared to the patients in the Norwegian Hip Fracture Register, which covers 86–94% of all hip fractures in Norway, the patients in our study had a mean age of 79.4 years and a mean ASA score of 2.5, compared to 80.0 years, and an ASA score of 2.7 in the register [47]. Our results are not generalizable for hip fracture patients from nursing homes, or patients with severe physical or cognitive impairment. However, the relatively robust participants in our study are more likely to benefit from intensive rehabilitation compared to frailer patients, as found by a study by Prestmo et al on the benefit of orthogeriatric care in frail versus fit hip fracture patients [48].

Increasing age was associated with sarcopenia and low grip strength, but not with low muscle mass, which is in agreement with previous research on healthy older people [49]. There is a significant association between low BMI and sarcopenia in other studies on sarcopenia in hip fracture patients [6, 8], which is consistent with the finding that BMI is negatively associated with sarcopenia, muscle mass and grip strength. Nutritional risk by NRS 2002 was associated with sarcopenia in unadjusted analysis, but not in adjusted analysis. This is explained by how NRS 2002 is scored, with higher scores for low BMI and age greater than 70 years. The NRS 2002 was developed as screening tool for identifying hospitalized patients likely to benefit from nutritional interventions, and is not primarily a tool to diagnose undernutrition. Albumin is a biomarker of undernutrition and is a risk factor for mortality in hip fracture patients [50]. In the present study, low albumin was associated with sarcopenia, low muscle mass and low grip strength. Serum albumin and vitamin D are reduced in inflammatory states [51] such as in hip fracture or surgery, and thus caution is warranted when interpreting this association. There are numerous studies describing important associations between serum albumin and clinical outcomes in patients with hip fracture, where serum albumin has been measured both before [52], and after surgery [53]. Visser et al [54] found an increased loss of muscle mass in participants with low serum albumin in older persons living in the community, which indicates a relevant association between sarcopenia and albumin in a setting without acute inflammation. The finding in this study that low BMI and serum albumin are associated with sarcopenia supports the hypothesis that nutrition and sarcopenia are associated and that nutritional interventions such as supplemental protein, specific amino acids or β-hydroxy-β-methylbutyrate can improve muscle mass, strength and physical performance [55–58]. The present study found no association between serum vitamin D and sarcopenia, which is in agreement with the study by Gonzalez-Montalvo et al [6] on sarcopenia in hip fracture patients. Participants in this study had higher levels of serum vitamin D compared to other studies on patients with hip fracture [59, 60], likely explained by the fact that half the participants used supplemental vitamin D before the fracture. The ASA score and the number of chronic diseases are predictors of mortality after hip fracture [26]. Polypharmacy or use of potentially inappropriate medication has been found to increase the risk of hip fracture [61], the risk of injurious falls after hip fracture [25], and reduced mobility and grip strength in hospitalized elderly [62]. In the present study, there was an association between higher ASA score, using more medications and sarcopenia, but no association between the Charlson index and sarcopenia. There is probably a causal relationship between comorbidities, polypharmacy and sarcopenia. Future studies should examine the effect on sarcopenia by reducing inappropriate polypharmacy [63]. The participants in the present study had low values on the Charlson index with a median score of one. Many common chronic conditions, such as hypertension, angina or osteoporosis are not counted as part of the Charlson index and it is possible that the index does not fully capture the burden of chronic diseases.

The associations found in the present study between sex and low muscle mass, low grip strength and reduced mobility, had wide confidence intervals. This indicates that the methods for determining muscle mass, grip strength and mobility and risk factors were insufficiently precise or that there were too few male participants with data on sarcopenia status in the study, only 50 out of 202. Men have a worse prognosis after hip fracture with a 4.6-fold increased mortality after hip fracture compared to 2.8- fold increase in women [10]. The reasons for this are unclear and at odds with other research that has found a greater prevalence of physical frailty in women [64]. Di Monaco et al [65] found that male patients with hip fracture were more likely to have low muscle mass using sex specific cut-points, and our results agree with this. Future studies should examine if low muscle mass in men with hip fracture can explain the excess mortality.

## Conclusion

Among previously ambulatory, community-living hip fracture patients, the prevalence of sarcopenia was 37%. Sarcopenia was positively associated with age, ASA score and polypharmacy, and negatively associated with BMI and albumin. By using anthropometry, grip strength and self-reported mobility it is feasible to determine sarcopenia at the bedside in postoperative hip fracture patients.
